# Targeting casein kinase 1 for cancer therapy: current strategies and future perspectives

**DOI:** 10.3389/fonc.2023.1244775

**Published:** 2023-11-08

**Authors:** Ngo Hoang Long, Sook-Jeong Lee

**Affiliations:** Department of Bioactive Material Sciences, Jeonbuk National University, Jeonju, Jeollabuk-do, Republic of Korea

**Keywords:** casein kinase 1, cancer therapy, clinical strategy, gene targeting, immunotherapy, RNA interference

## Abstract

Casein Kinase 1 (CK1) is a family of serine/threonine protein kinases that play a crucial role in various cellular processes, including cell proliferation, survival, and metabolism. The dysregulation of CK1 expression has been implicated in the development and progression of several types of cancer, making it an attractive target for anticancer therapy. In this review, we provide an overview of the current strategies employed to target CK1 for cancer therapy and discuss the future perspectives in this field. We highlight the different approaches, including small molecule inhibitors, RNA interference, genome editing, and immunotherapies, which hold immense potential for targeted modulation of CK1 activity in cancer cells. Furthermore, we discuss the challenges associated with targeting CK1 and propose potential strategies to overcome these hurdles. Overall, targeting CK1 holds great promise as a therapeutic strategy for cancer treatment, and further research in this area is warranted.

## 1 Introduction

Casein kinase 1 (CK1) has emerged as a promising target for cancer therapy, with growing evidence linking its altered expression and activity to carcinogenesis and cancer progression (1–6). CK1 belongs to a distinct subgroup of the family of Ser/Thr protein kinases, comprising six isoforms in humans: CK1α, γ1, γ2, γ3, δ, and ϵ. It functions as an autonomous monomeric enzyme, independent of cofactors, and its activity can be regulated by the autophosphorylation of carboxyterminal residues (7, 8).

CK1 plays a crucial role in various cellular processes, including the regulation of cell, survival/apoptosis signaling pathways, and cell division (9). Notably, CK1 isoforms have been associated with cell cycle progression (10), chromosome segregation (11), apoptosis (12), DNA repair (13), the circadian rhythm (14), ribosome biogenesis (15), vesicle trafficking (16), and modulation of signaling pathways such as p53 (7), Wnt, Shh (5), and Hippo (17). Recent advances in our understanding of CK1 have shed light on its involvement in protein phosphorylation processes and its impact on signaling pathways mediated by the epidermal growth factor receptor, estrogen receptor, G protein-coupled estrogen receptor (10). Targeting CK1 holds significant promise as a therapeutic strategy for cancer treatment.

The aim of this review is to provide a comprehensive overview of the current strategies employed in targeting CK1 for cancer therapy. It will explore different approaches, including small-molecule inhibitors, RNA interference (RNAi), and immunotherapies. Additionally, it will discuss the challenges associated with the targeting of CK1 and highlight future perspectives in this field. By elucidating the current state of CK1-targeted cancer therapies and outlining potential future directions, this review aims to contribute to the advancement of effective and innovative strategies for cancer treatment.

## 2 CK1 isoforms and their roles in cancer

**Figure 1:**
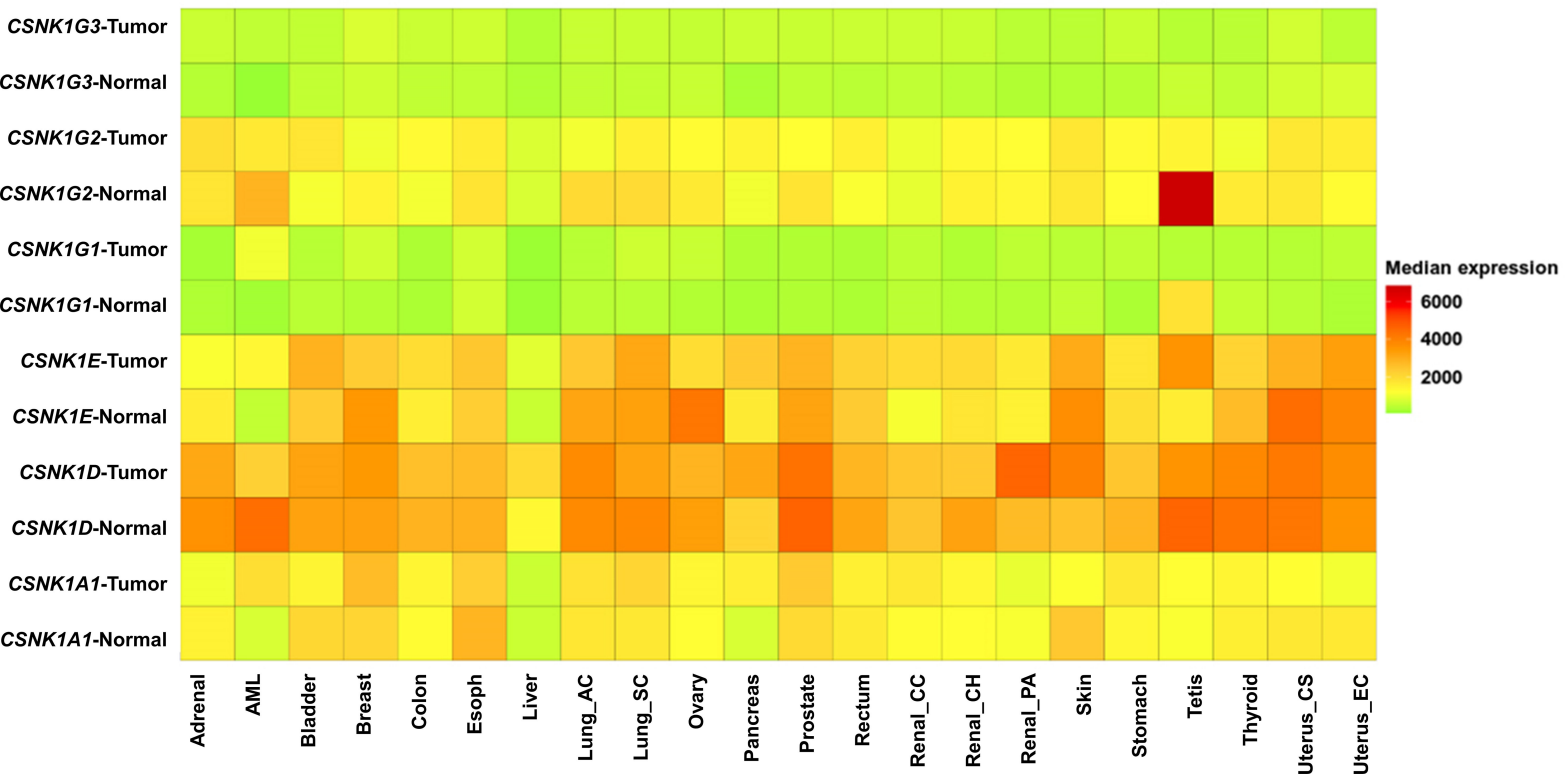
Heatmap comparing gene expression in normal human tissues and matched tumor tissues of various cancer types, including adrenal, acute myeloid leukemia (AML), bladder, breast, colon, esophagus, liver, lung adenocarcinoma (lung_ac), lung squamous cell carcinoma (lung_sc), ovary, pancreas, prostate, rectum, renal clear cell carcinoma (renal_cc), renal chromophobe (renal_ch), renal papillary carcinoma (renal_pa), skin, stomach, testis, thyroid, uterus carcinosarcoma (uterus_cs), and uterus endometrioid carcinoma (uterus_ec). Gene expression data was generated from GTEx, TCGA, and TARGET databases using TNMplot (18). The heatmap represents relative gene expression levels, with higher expression indicated by warmer colors and lower expression by cooler colors.

CK1 isoforms, including CK1α, CK1δ, CK1ϵ, and CK1γ, play diverse roles in cancer development and progression (7) (**Figure 1**). Dysregulation of these isoforms contributes to various cancer-related processes, including Wnt/β-catenin signaling, disruption of the circadian rhythm, DNA repair, cell cycle progression, and stability of oncogenic proteins. Targeting specific CK1 isoforms represents a promising avenue for the development of novel anti-cancer agents.

### 2.1 CK1α

**Figure 2:**
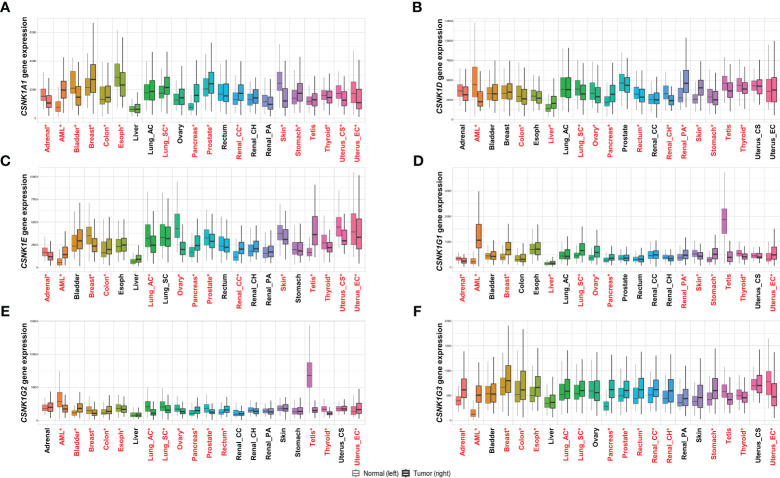
Comparison of the expression of each casein kinase (CKN) isoform encoded gene in humans, including **(A)** *CNSK1A1*, **(B)** *CSNK1D*, **(C)** *CNSK1E*, **(D)** *CSNK1G1*, **(E)** *CNSK1G2*, and **(F)** *CNSK1G3*, in normal and tumor tissues of various cancer types, including adrenal, acute myeloid leukemia (AML), bladder, breast, colon, esophagus, liver, lung adenocarcinoma (lung_ac), lung squamous cell carcinoma (lung_sc), ovary, pancreas, prostate, rectum, renal clear cell carcinoma (renal_cc), renal chromophobe (renal_ch), renal papillary carcinoma (renal_pa), skin, stomach, testis, thyroid, uterus carcinosarcoma (uterus_cs), and uterus endometrioid carcinoma (uterus_ec). Gene expression data was obtained from the GTEx, TCGA, and TARGET databases using TNMplot (18). Significant differences are identified by Mann-Whitney U test are denoted by red asterisks (*P<0.05).

CK1α is one of the most extensively studied isoforms of CK1 and has been implicated in several malignancies (**Figure 2A**). It plays a critical role in regulating the Wnt/β-catenin signaling pathway, which is frequently dysregulated in cancer. CK1α phosphorylates β-catenin, leading to its degradation and inhibition of downstream Wnt signaling. Dysregulation of CK1α in cancer leads to abnormal accumulation of β-catenin and activation of Wnt signaling, thus facilitating tumor growth and metastasis (5). CK1α has been shown to play a role in the regulation of the immune response in cancer. CK1α expression is altered in response to interferon-γ (19) and is required for the expression of pseudo-Programmed death-ligand 1 (20, 21), an immune checkpoint protein that is often overexpressed in cancer cells to evade immune surveillance (22).CK1α has also been implicated in the regulation of DNA damage response pathways in cancer cells (23, 24) and in mouse embryonic stem cells (25), suggesting a potential role in promoting genomic instability.

### 2.2 CK1δ and CK1ϵ

CK1δ and CK1ϵ are closely related isoforms that share a high degree of sequence homology. Both isoforms have been associated with carcinogenesis and cancer progression (**Figures 2B, C** **)**. CK1δ and CK1ϵ participate in the regulation of the circadian rhythm, and the disruption of circadian clock genes has been linked to an increased risk of cancer. Dysregulated CK1δ and CK1ϵ activity can perturb the normal circadian rhythm, leading to disturbances in cellular processes, including DNA repair, cell cycle progression, and apoptosis, which contribute to tumorigenesis.

CK1δ continues to be an important target for cancer therapy, particularly in triple-negative breast cancer (26, 27). Rosenberg et al. demonstrated that *CSNK1D* was overexpressed in human breast cancers and established a link between CK1δ and Wnt/β-catenin signaling pathway, whereby inhibition CK1δ blocked the localization of β-catenin and the transcriptional activity of T cell factor, resulting in the apoptosis of breast cancer cells (26). In colorectal cancer, CK1 and expression of CK1δ and ε are altered compared to the normal bowel epithelium, and high CK1ε expression is significantly correlated with prolonged patient survival. Mutations within exon 3 of CK1δ have also been detected in colorectal tumors (28). CK1ε has also been implicated in the regulation of immune responses in cancer. The study by Zhou et al. found that CK1ε interacted with and phosphorylated TRAF3, which promoted the production of type I interferon, a key component of the antiviral immune response. The phosphorylation of TRAF3 by CK1 ε led to the recruitment of another kinase called TBK1 to TRAF3, suggesting that CK1 plays a significant role in regulating the innate immune response to viral infections and provides a novel mechanism of immunoregulation (29). CK1ε has also been shown to play a role in the regulation of the Hippo signaling pathway, a key pathway involved in the regulation of cell proliferation and apoptosis. Its aberrant expression has been identified in a variety of cancers, including breast and ovarian cancers (7, 30). CK1ε plays a crucial role in tumor cell proliferation via interacting and phosphorylating 4E-BP1 at T41 and T50, which is essential for 4E-BP1 inactivation, increasing mRNA translation, and cell proliferation (31). Moreover, CK1δ and CK1ϵ have been implicated in modulating the stability and activity of key oncogenic proteins, such as p53, c-Myc, and NF-κB, further highlighting their roles in cancer biology.

### 2.3 CK1γ

CK1γ1,2,3 are less well-characterized isoforms compared to CK1α, CK1δ, and CK1ϵ. However, emerging evidence suggests its involvement in cancer-related processes (9, 32). The role of CK1γ in cancer remains unclear, although recent studies have suggested a potential role in the regulation of autophagy (9), a process by which cells recycle damaged or unwanted components. CK1γ has been implicated in the regulation of cell cycle progression and mitotic spindle formation, which are crucial for maintaining genomic stability. Dysregulation of CK1γ has been observed in different types of cancer, including breast (9) and lung cancer (4) (**Figures 2D–F**). CK1γ has also been shown to regulate the activity of the transcription factor NF-κB, a key regulator of immune responses, suggesting a potential role in the regulation of immune surveillance in cancer.

Understanding the specific roles of each CK1 isoform in cancer is essential for the development of isoform-specific therapeutic strategies. Targeting individual isoforms can help modulate key signaling pathways, restore circadian rhythm, and disrupt interactions with oncogenic proteins, thus inhibiting tumor growth and metastasis. However, additional research is needed to elucidate the precise functions of the CK1 isoforms in diverse types of cancer and to develop isoform-specific inhibitors with optimal efficacy and safety profiles. Validating the potency of specific CK1 isoforms in defined tumor types will be crucial in the future, as their roles can vary and can sometimes exert opposite effects. This validation will enhance our understanding and enable the development of targeted therapies tailored to specific tumors.

## 3 Small molecule inhibitors targeting CK1

### 3.1 Development of CK1-specific inhibitors

The development of CK1-specific inhibitors has been driven by the need for isoform-selective targeting to maximize therapeutic efficacy and minimize off-target effects. High-throughput screening, studies of structure-activity relationships, and virtual screening techniques have facilitated the identification and optimization of small molecules with high affinity and selectivity for CK1 isoforms. Several chemical scaffolds, including arylimidazoles, benzimidazoles, benzothiazoles, isoxazoles, purines, pyrazoles, BTX-A51, CKI-7, D4476, IC261, pyrido[3,4-g]quinazoline, PF-4800567, PF-670462, and TG003, have been explored as CK1 inhibitors (33).

### 3.2 Mechanisms of action

Small-molecule inhibitors targeting CK1 exert their effects through multiple mechanisms. The dominant molecular and cellular mechanisms that contribute to the effects of CK1 inhibition in preclinical models and clinical trials have not been fully elucidated. These mechanisms encompass both cell-intrinsic processes and intricate interactions with the tumor microenvironment and cell-to-cell communication. CK1 serves as a critical regulator of various signaling pathways, including Wnt, Hedgehog, and Yap/Taz (5, 7, 8, 34). A comprehensive understanding of these mechanisms will provide valuable insights into the therapeutic implications of CK1 inhibition and will pave the way for the development of targeted interventions in cancer therapy. A common mechanism is the direct inhibition of CK1 kinase activity by binding to the ATP-binding pocket of the kinase domain (35). These inhibitors compete with ATP for binding, thus preventing the phosphorylation of CK1 substrates. Another mechanism involves allosteric modulation of CK1 activity by binding to distinct sites outside the ATP-binding pocket, inducing conformational changes that inhibit kinase activity (36). Additionally, some inhibitors promote protein degradation by targeting specific CK1 substrates, leading to the down-regulation of oncogenic signaling pathways.

### 3.3 Efficacy in clinical studies

In the clinical setting, the development of CK1 inhibitors is still in its preliminary stages. Despite the absence of selective CK1 inhibitors in clinical trials, umbralisib, a dual inhibitor of PI3Kδ and CK1ϵ, is currently being evaluated in clinical trials for patients with chronic lymphocytic leukemia (CLL) (37) and non-Hodgkin lymphomas (NHL) (38). CK1ϵ has been observed to be upregulated in CLL, and inhibition of CK1ϵ has been demonstrated to prevent chemotaxis of cancer cells in CLL (39). Additionally, CK1ϵ inhibition has also displayed encouraging results in preclinical investigations involving various hematological malignancies, such as NHL, myelodysplastic syndrome (MDS), acute myeloid leukemia (AML), and multiple myeloma (MM) (40). The activation of the p53 pathway by inhibition of CK1α has shown promising preclinical activities in MDS, AML, and MM, leading to the establishment of clinical trials for the first CK1α inhibitor (BTX-A51, NCT04243785). The results from these studies will provide valuable insights into the therapeutic potential of CK1 inhibitors in a clinical setting.

### 3.4 Limitations of small molecule inhibitors

Inhibitor resistance is a common challenge in cancer treatment, including kinase inhibitors (KIs) (41). Mutations that impair drug binding or interfere with necessary conformational changes can lead to resistance to KIs. For example, the T315I mutation confers resistance to certain Bcr-Abl tyrosine kinase inhibitors (TKIs) (42). To overcome resistance, third-generation TKIs like ponatinib have been developed, but they may have safety concerns (43, 44). Dysregulation of signaling pathways is a common mechanism contributing to drug resistance. Phospho-proteomics analysis has revealed that compensatory pathways, such as epithelial-to-mesenchymal transition and cell adhesion, contribute to resistance in hematological malignancies patients treated with multi-KIs sorafenib (45). Distinct types of inhibitors target specific binding sites, such as type III (within ATP pocket) and type IV (substrate-binding domain) inhibitors, and physiological mechanisms of kinases. These inhibitors have been approved by the FDA for cancer treatment (41).

## 4 RNA interference-based approaches and genome editing technologies

RNA interference (RNAi) has emerged as a powerful tool for silencing specific genes, including *CSNK1*, offering a potential therapeutic avenue for cancer treatment. RNAi-based approaches utilize small RNA molecules, such as small interfering RNAs (siRNAs) or short hairpin RNAs (shRNAs), to specifically target and degrade *CSNK1* mRNA, thereby reducing the expression of CK1 protein.

The application of RNAi-based approaches to inhibit *CSNK1* has several advantages. First, it enables precise and selective targeting of *CSNK1*, allowing isoform-specific down-regulation. This isoform specificity is crucial, as different *CSNK1* isoforms may have distinct functions and contributions to tumorigenesis in diverse types of cancer (**Figure 2**). By selectively silencing specific isoforms, RNAi-based strategies can effectively disrupt CK1-mediated signaling pathways implicated in cancer progression (46). Second, RNAi-based approaches offer the potential for systemic administration and broad delivery to various tumor sites. The development of efficient delivery systems, such as nanoparticles or viral vectors, has improved the delivery and stability of RNAi molecules, enabling their effective transport to tumor cells (47, 48). This systemic delivery approach enhances the feasibility and applicability of RNAi-based therapeutics for the inhibition of *CSNK1* in cancer treatment (49). Moreover, the combinatory potential of RNAi-based approaches with other treatment modalities holds promise for enhanced therapeutic efficacy. By targeting *CSNK1* along with other key signaling molecules or pathways, synergistic effects may be achieved, leading to more profound and sustained anti-tumor responses. Additionally, combining RNAi-based approaches with conventional chemotherapy, radiotherapy, or targeted therapies will potentially overcome drug resistance and improve treatment outcomes.

However, several challenges need to be addressed to fully exploit the potential of RNAi-based approaches for the inhibition of *CSNK1* in cancer therapy. Efficient delivery of RNAi molecules to tumor cells while minimizing off-target effects remains a critical hurdle. Further optimization of delivery systems, including improving stability, cellular uptake, and intracellular localization, is necessary. Furthermore, the identification of reliable and predictive biomarkers for patient stratification and monitoring of treatment response is essential for the successful implementation of RNAi-based therapies. Biomarkers that can indicate CK1 expression levels, isoform-specific alterations, or downstream pathway activity will facilitate personalized treatment strategies and improve patient outcomes.

In addition to RNAi-based approaches, the use of genome editing technologies, such as CRISPR/Cas9, holds immense potential for targeted modifications of the *CSNK1* gene. The remarkable efficiency and simplicity of CRISPR/Cas9 enable precise DNA cleavage at specific sites within the *CSNK1* gene, facilitating the manipulation of CK1 expression and function with high precision. By introducing double-stranded breaks, CRISPR/Cas9 enables targeted gene disruption, knockout, or insertion of specific sequences to modulate *CSNK1* expression or function (50). Similarly, zinc finger nucleases (ZFNs), transcription-activator-like effector nucleases (TALENs) (51), Homing Endonucleases (52), and CRISPR-Cpf1 (53) offer alternative platforms for site-specific DNA cleavage and gene editing, allowing precise modifications of the *CSNK1* gene.

These genome editing technologies will provide opportunities to investigate the functional consequences of *CSNK1* modulation and uncover the intricate roles of CK1 in cancer development and progression. By introducing specific mutations or alterations in *CSNK1*, researchers will be able to elucidate the impact of these modifications on downstream signaling pathways and cellular processes involved in cancer. In addition, these techniques will enable the exploration of potential therapeutic targets and the development of personalized treatment strategies based on specific genetic alterations of *CSNK1*.

## 5 Immunotherapies targeting CK1

Although the roles of CK1 in immunology are not yet fully understood, it is increasingly recognized that CK1 plays a significant role in the modulation of immune responses, particularly through its involvement in the Wnt signaling pathway (7, 29, 40, 54, 55). This emerging knowledge presents an exciting opportunity to explore the potential of developing immunotherapeutic approaches targeting CK1.

The Wnt signaling pathway is known to be critical in various aspects of the regulation of immune responses, including T cell development, differentiation, and activation (56–58). CK1, as a key regulator of the Wnt pathway, influences immune cell function and can impact the outcome of immune responses (59). Therefore, investigating the precise role of CK1 in immune regulation and exploring its potential as a target for immunotherapy holds great promise. Immunotherapies targeting CK1 could offer novel strategies to modulate immune responses in the context of cancer and other immune-related diseases (39). By selectively targeting CK1, it may be possible to manipulate the Wnt signaling pathway and enhance immune cell activation or suppress immune tolerance mechanisms, leading to improved anti-tumor or immune modulatory effects.

Immunotherapies targeting CK1 have several advantages. They have high specificity, allowing for selective targeting of CK1 overexpressing tumors while sparing healthy tissues. Immunotherapies can also elicit potent and durable anti-tumor responses, providing long-term control of cancer progression. In addition, they offer the possibility of systemic treatment, enabling the eradication of both primary and metastatic tumors. However, several challenges need to be addressed to optimize immunotherapies targeting CK1. One key challenge is identifying reliable biomarkers that accurately predict CK1 expression or isoform-specific alterations in tumors. These biomarkers will aid in patient selection, treatment stratification, and monitoring of therapy response. Additionally, overcoming the immunosuppressive tumor microenvironment is crucial to achieving successful immunotherapy. Strategies to improve T cell infiltration, modulate immune checkpoints, or reprogram the tumor microenvironment to promote immune responses are actively being explored.

Additional research is needed to elucidate the specific functions of CK1 in immune cells and to understand its intricate involvement in the regulation of the immune response. This knowledge will guide the development of innovative immunotherapeutic approaches, such as CK1 inhibitors or CK1-specific antibodies, which can selectively modulate CK1 activity in immune cells.

## 6 Challenges and future perspectives

One major challenge is the isoform-specific targeting of CK1. As different CK1 isoforms can have distinct functions and contributions to tumorigenesis, it is crucial to develop strategies that selectively inhibit isoforms involved in relevant to specific types of tumors. This requires a deeper understanding of the isoform-specific roles of CK1 in different cancers and the development of selective inhibitors or modulators for each isoform. Another challenge lies in the development of effective delivery systems for CK1-targeted therapies. Efficient delivery to tumor cells while minimizing off-target effects is critical to therapeutic success. Further advancements in delivery technologies, such as the development of nanoparticle-based systems or targeted drug delivery approaches, are necessary to ensure the precise and effective delivery of CK1 inhibitors to tumor tissues.

Combination therapies involving CK1 inhibitors or immunotherapies with other treatment modalities can enhance therapeutic efficacy and overcome resistance mechanisms. Combining CK1-targeted therapies with conventional chemotherapy, radiotherapy, immune checkpoint inhibitors, or other targeted agents can synergistically inhibit multiple signaling pathways and improve anti-tumor immune responses. Rational design and optimization of combination regimens that rely on mechanistic insights and preclinical studies will be crucial to harnessing the full potential of CK1-targeted therapies in the clinic.

The identification of predictive biomarkers is essential for optimal patient selection and treatment monitoring in CK1-targeted therapies. Biomarkers can help stratify patients who are most likely to benefit from CK1 inhibitors or immunotherapies and provide information on response and treatment resistance. Potential biomarkers may include the determination of CK1 expression, isoform-specific signatures, or alterations in downstream signaling molecules. The development of robust and reliable biomarkers through comprehensive genomic profiling, proteomic analyses, or non-invasive imaging techniques will facilitate personalized treatment strategies and improve patient outcomes.

Looking ahead, the future perspectives of targeting CK1 for cancer therapy appear to be promising. Continued research efforts are required to deepen our understanding of the intricate mechanisms underlying CK1 dysregulation in cancer and its crosstalk with other signaling pathways. This will enable the identification of new therapeutic targets and the development of innovative strategies to overcome treatment resistance and improve patient outcomes.

## 7 Conclusions

While the pursuit of CK1 as a therapeutic target in cancer therapy holds great promise, it confronts noteworthy challenges. A pivotal obstacle lies in the imperative need for isoform-specific targeting of CK1. Given the divergent functionalities of distinct CK1 isoforms and their variable contributions to tumorigenesis, the development of strategies that can selectively inhibit isoforms relevant to specific tumor types becomes imperative. Moreover, the specter of undesired side effects, off-target repercussions, and the emergence of drug resistance looms as potential impediments in CK1-targeted therapy. In response to these challenges, RNAi-based and genome editing approaches emerge as valuable strategies. These methodologies have demonstrated their utility by enriching our comprehension of the pathological intricacies pervasive across various malignancies, and they have excelled in uncovering novel drug targets and elucidating the molecular underlying the adaptive responses following kinase inhibition. Although it is undeniably formidable, with continued research and development, the potential of CK1-targeted therapies can be harnessed to improve patient outcomes and contribute to the evolving landscape of cancer therapies.

## Author contributions

LNH: conceptualization, investigation, and writing – original draft. S-JL: project administration, resources, supervision, and writing – review & editing. All authors contributed to the preparation of the article and have approved the submitted version.
